# Clinical hepatic indices serve as predictive markers for sinusoidal obstruction syndrome after allogeneic HSCT

**DOI:** 10.1007/s12185-026-04191-5

**Published:** 2026-03-16

**Authors:** Hiroya Ichikawa, Kimikazu Yakushijin, Yumiko Inui, Naoko Takemoto, Takahiro Tsuji, Kotaro Iida, Sakura Kamido, Isamu Harima, Yuri Okazoe-Hirakawa, Sakuya Matsumoto, Rina Sakai, Keiji Kurata, Akihito Kitao, Yasuyuki Saito, Shinichiro Kawamoto, Katsuya Yamamoto, Mitsuhiro Ito, Tohru Murayama, Hiroshi Matsuoka, Hironobu Minami

**Affiliations:** 1 Division of Medical Oncology and Hematology, Department of Medicine, Kobe University Hospital and Graduate School of Medicine, Kobe, Hyogo Japan; 2 Transfusion Medicine and Cell Therapy, Kobe University Hospital and Graduate School of Medicine, Kobe, Hyogo Japan; 3 Laboratory of Hematology, Division of Medical Biophysics, Kobe University Graduate School of Health Sciences, Kobe, Hyogo Japan; 4 Department of Hematology, Hyogo Cancer Center, Akashi, Hyogo Japan; 5 Department of Integrated Analyses of Bioresource and Health Care, Kobe University Graduate School of Medicine, Kobe, Hyogo Japan

**Keywords:** SOS/VOD, Hepatic indices, Allogeneic transplantation

## Abstract

Prediction and early diagnosis are critical for the effective treatment of sinusoidal obstruction syndrome/veno-occlusive disease (SOS/VOD) after hematopoietic stem cell transplantation (HSCT). This study aimed to investigate the utility of clinical hepatic indices easily calculated in routine practice, namely the Endothelial Activation and Stress Index (EASIX), aspartate aminotransferase-to-platelet ratio index (APRI), Fibrosis-4 (FIB-4), reversed Albumin-Bilirubin grade (rALBI), Model for End-Stage Liver Disease (MELD), and MELD score and the serum sodium concentration (MELDNa). We retrospectively analyzed longitudinal clinical data from 175 allogeneic HSCTs at our institution. The 22 patients who eventually developed clinical SOS/VOD were used as the reference standard. At baseline, EASIX, APRI, FIB-4, rALBI, MELD, and MELDNa were analyzed using the receiver operating characteristic method, with resulting area under the curve (95% confidence interval) of 0.739 (0.607–0.871), 0.770 (0.641–0.898), 0.760 (0.645–0.875), 0.672 (0.530–0.815), 0.577 (0.440–0.713), and 0.642 (0.449–0.784), respectively. After HSCT, these indices were also associated with SOS/VOD, even 7 days before diagnosis (all *p*-values < 0.001). In conclusion, clinical hepatic indices are useful for prediction and early diagnosis of SOS/VOD in allogeneic HSCT recipients. Further studies are required to determine their optimal clinical application.

**Supplementary Information:**

The online version contains supplementary material available at 10.1007/s12185-026-04191-5.

## Introduction

Sinusoidal obstruction syndrome/veno-occlusive disease (SOS/VOD) following hematopoietic stem cell transplantation (HSCT) is a clinically substantial complication owing to its high mortality rate, particularly in severe cases [1–3]. Defibrotide is the only approved drug for SOS/VOD and has a potential to improve outcomes in patients with poor prognosis [4]. Early initiation of defibrotide is essential for effective treatment [5, 6]; accordingly, predicting and diagnosing SOS/VOD early is clinically important. In 2023, the European Society for Blood and Marrow Transplantation (EBMT) refined the diagnostic and severity criteria for SOS/VOD (refined EBMT criteria 2023), introducing a new diagnostic category, “probable SOS/VOD,” to facilitate earlier diagnosis [7]. We previously reported that the “probable SOS/VOD” criteria enabled us to diagnose SOS/VOD a median of 5 days earlier than the conventional criteria [8].

The Endothelial Activation and Stress Index (EASIX) can be easily calculated from serum creatinine, lactate dehydrogenase, and platelet counts and has recently gained attention in HSCT [9, 10]. EASIX is assumed to represent endothelial injury, a key cause of HSCT-specific complications, particularly SOS/VOD and transplant-associated thrombotic microangiopathy [11]. Some researchers have demonstrated that the baseline EASIX value is a useful predictive marker of SOS/VOD [12, 13]. However, after HSCT, the utility of EASIX is not sufficiently understood, particularly in the impending phase of SOS/VOD.

Several similar indices have been widely used in the field of hepatic diseases, such as aspartate aminotransferase-to-platelet ratio index (APRI) [14], Fibrosis-4 (FIB-4) [15], Albumin-Bilirubin grade (ALBI) [16], Model for End-Stage Liver Disease (MELD) [17, 18], and MELD score and the serum sodium concentration (MELDNa) [19]. Similar to the EASIX, all indices can be calculated rapidly and easily using routine blood test values. The utility of these indices in SOS/VOD has not been sufficiently investigated.

Understanding these indices will aid physicians in their appropriate utilization, leading to improved SOS/VOD treatment. Therefore, we evaluated the utility of the clinical hepatic indices in predicting SOS/VOD during the clinical course of allogeneic HSCT.

## Methods

### Patients

We retrospectively collected and analyzed longitudinal clinical data of allogeneic HSCT patients during admission and visit days from the initiation of conditioning. Data from patients aged ≥ 18 years who underwent allogeneic HSCT at Kobe University Hospital between January 2012 and December 2023 were analyzed. We used available data at the most recent time point (a maximum of 7 days) before the initiation of conditioning, as baseline data. This study was approved by the Ethics Committee of Kobe University Hospital. Patients who participated in this study were offered the opportunity to opt out. This study was conducted in accordance with the principles of the Declaration of Helsinki.

### Definitions

The formulas for calculating the clinical hepatic indices are described in Fig. S1. In this study, the sign of ALBI was reversed for convenience of analysis (as “reversed ALBI [rALBI]”). When required data for calculation were missing, retroactive data substitution was allowed within 7 days (except for the prothrombin time-international normalized ratio, which allowed substitution within 21 days). SOS/VOD diagnosis was based on the refined EBMT criteria 2023 [7], regarding “clinical SOS/VOD” as the reference standard and excluding patients who received alternative diagnoses through biopsy or autopsy. Conditioning regimens were classified as myeloablative if any of the following were used: total body irradiation > 8 Gy, intravenous busulfan > 7.2 mg/kg, or melphalan > 140 mg/m^2^ [20]. All others were classified as reduced-intensity conditioning. Transplantation-related mortality (TRM) was defined as death other than the primary disease.

### Matching analysis

To conduct a comparative investigation of the post-HSCT situation, we extracted appropriate controls for each SOS/VOD case. We applied the nested case–control method [21] and sampled control cases from the non-SOS/VOD group, maintaining a ratio of 1:2 for SOS/VOD cases to controls. We adopted age, sex, conditioning type (myeloablative or reduced-intensity), presence of human leukocyte antigen genotype mismatch, and hematopoietic cell transplantation-specific comorbidity index as adjusting factors. Resampling identical cases was not permitted. In each matched-pair group, we collected clinical data from the day of SOS/VOD diagnosis for the case and from the same post-HSCT day for the corresponding control.

### Statistical analysis

Categorical and continuous variables of patient characteristics were compared using the Fisher exact test and Mann–Whitney U test, respectively. We compared the objective values between the SOS/VOD cases and controls using the exact Wilcoxon rank-sum test. The proportion of SOS/VOD incidence was compared using the Fisher exact test. Overall survival was estimated using the Kaplan–Meier method and log-rank test. TRM was estimated using the Fine–Gray method, considering relapse-related death as a competing risk. To determine the most appropriate cutoff threshold for each index, we constructed a receiver operating characteristic (ROC) curve and identified the point closest to the upper-left corner as the threshold. The area under the curve (AUC) for the ROC curve was compared using the DeLong test. Statistical significance was defined as a two-tailed p-value < 0.05. All analyses were performed using R version 4.4.1 and Visual Basic for Applications with Microsoft Excel.

## Results

### Patient characteristics

We analyzed a total of 175 cases (involving 153 patients) and diagnosed 25 cases of “clinical SOS/VOD.” Of the 25 cases, three were histologically confirmed as having other diseases (one herpes simplex virus infection and two chronic graft-versus-host diseases), leaving 22 cases as the reference standard. The patient characteristics are summarized in Tables 1 and S1. Among the reference standards, “clinical SOS/VOD” was diagnosed at a median of 23.5 days (interquartile range 12.5–68.75) after HSCT. Haploidentical related-HSCT was performed in one case in the entire cohort. Ursodeoxycholic acid was routinely used for SOS/VOD prophylaxis at our hospital, at the discretion of the physicians. The median follow-up period was 1,067.5 days (range 33–4018) after HSCT for the 106 survivors. All patients underwent daily blood tests at least until engraftment, and the percentage of missing laboratory data was 11.1% between days 0 and 30 after HSCT, without considering data substitution.

**Table 1:** Patient characteristics

	Non-SOS/VOD	SOS/VOD	Total cases	P-value	Std. diff
N	153	22	175		
Age, median (range)	52 (19–69)	52 (28–65)	52 (19–69)	0.379	0.149
Sex (%)				0.819	0.073
Male/Female	89/64 (58.2/41.8)	12/10 (54.5/45.5)	101/74 (57.7/42.3)		
Primary disease (%)				0.500	0.533
AML	60 (39.2)	5 (22.7)	65 (37.1)		
ALL/LBL	31 (20.3)	4 (18.2)	35 (20.0)		
MDS	18 (11.8)	4 (18.2)	22 (12.6)		
CML/MPN	10 (6.5)	2 (9.1)	12 (6.9)		
ML	13 (8.5)	2 (9.1)	15 (8.6)		
MM	4 (2.6)	0 (0.0)	4 (2.3)		
ATLL	7 (4.6)	2 (9.1)	9 (5.1)		
AA	2 (1.3)	1 (4.5)	3 (1.7)		
Others*	8 (5.2)	2 (9.1)	10 (5.7)		
Disease status (%)				0.011	0.807
CR/PR/SD/PD/Unevaluable	102/15/2/27/7 (66.7/9.8/1.3/17.6/4.6)	7/3/0/10/2 (31.8/13.6/0.0/45.5/9.1)	109/18/2/37/9 (62.3/10.3/1.1/21.1/5.1)		
ECOG PS (%)				< 0.001	0.834
0/1/2/3/4	34/110/7/1/1 (22.2/71.9/4.6/0.7/0.7)	3/11/3/3/2 (13.6/50.0/13.6/13.6/9.1)	37/121/10/4/3 (21.1/69.1/5.7/2.3/1.7)		
HCT-CI (%)				0.158	0.484
0/1–2/ ≥ 3	41/33/79 (26.8/21.6/51.6)	2/5/15 (9.1/22.7/68.2)	43/38/94 (24.6/21.7/53.7)		
Source (%)				0.627	0.220
BM/PBSC/CB	63/26/64 (41.2/17.0/41.8)	10/5/7 (45.5/22.7/31.8)	73/31/71 (41.7/17.7/40.6)		
Donor (%)				0.551	0.144
Related/Unrelated	26/127 (17.0/83.0)	5/17 (22.7/77.3)	31/144 (17.7/82.3)		
Number of transplantation (%)				1.000	0.169
1st/2nd/3rd	128/23/2 (83.7/15.0/1.3)	19/3/0 (86.4/13.6/0.0)	147/26/2 (84.0/14.9/1.1)		
HLA serotype mismatch (%)				0.495	0.190
Yes/No	84/69 (54.9/45.1)	10/12 (45.5/54.5)	94/81 (53.7/46.3)		
HLA genotype mismatch (%)				0.488	0.205
Yes/No	92/61 (60.1/39.9)	11/11 (50.0/50.0)	103/72 (58.9/41.1)		
Conditioning (%)				0.644	0.127
MAC/RIC	60/93 (39.2/60.8)	10/12 (45.5/54.5)	70/105 (40.0/60.0)		
BU-containing regimen (%)				1.000	0.036
Yes/No	53/100 (34.6/65.4)	8/14 (36.4/63.6)	61/114 (34.9/65.1)		
TBI-containing regimen (%)				0.721	0.077
Yes/No	136/17 (88.9/11.1)	19/3 (86.4/13.6)	155/20 (88.6/11.4)		
GVHD prophylaxis (%)				0.245	0.368
TAC + MMF	129 (84.3)	17 (77.3)	146 (83.4)		
CyA + MMF	14 (9.2)	3 (13.6)	17 (9.7)		
TAC alone	9 (5.9)	1 (4.5)	10 (5.7)		
PTCY including	1 (0.7)	0 (0.0)	1 (0.6)		
Other	0 (0.0)	1 (4.5)	1 (0.6)		
Prior exposure to GO (%)	1 (0.7)	0 (0.0)	1 (0.6)	1.000	0.115
Prior exposure to INO (%)	3 (2.0)	0 (0.0)	3 (1.7)	1.000	0.200
Medication of UDCA (%)	148 (96.7)	21 (95.5)	169 (96.6)	0.559	0.066

SOS/VOD, sinusoidal obstruction syndrome/veno-occlusive disease; std. diff., standardized difference; AML, acute myeloid leukemia; ALL/LBL, acute lymphoblastic leukemia/lymphoma; MDS, myelodysplastic syndrome; CML/MPN, chronic myeloid leukemia/myeloproliferative neoplasm; ML, malignant lymphoma; MM, multiple myeloma; ATLL, adult T-cell leukemia/lymphoma; AA, aplastic anemia; CR, complete remission; PR, partial remission; SD, stable disease; PD, progressive disease; ECOG PS, Eastern Cooperative Oncology Group performance status; HCT-CI, hematopoietic cell transplant-specific comorbidity index; BM, bone marrow; PBSC, peripheral blood stem cell; CB, cord blood; HLA, human leukocyte antigen; MAC, myeloablative conditioning; RIC, reduced-intensity conditioning; BU, busulfan; TBI, total body irradiation; GVHD, graft-versus-host disease; TAC, tacrolimus; MMF, mycophenolate mofetil; CyA, cyclosporine; PTCY, posttransplantation cyclophosphamide; GO, gemtuzumab ozogamicin; INO, inotuzumab ozogamicin; UDCA, ursodeoxycholic acid

^*^The “others” disease category is composed of T-cell prolymphocytic leukemia, chronic active Epstein–Barr virus disease, etc.

### Utility of clinical hepatic indices at baseline

**Fig. 1:**
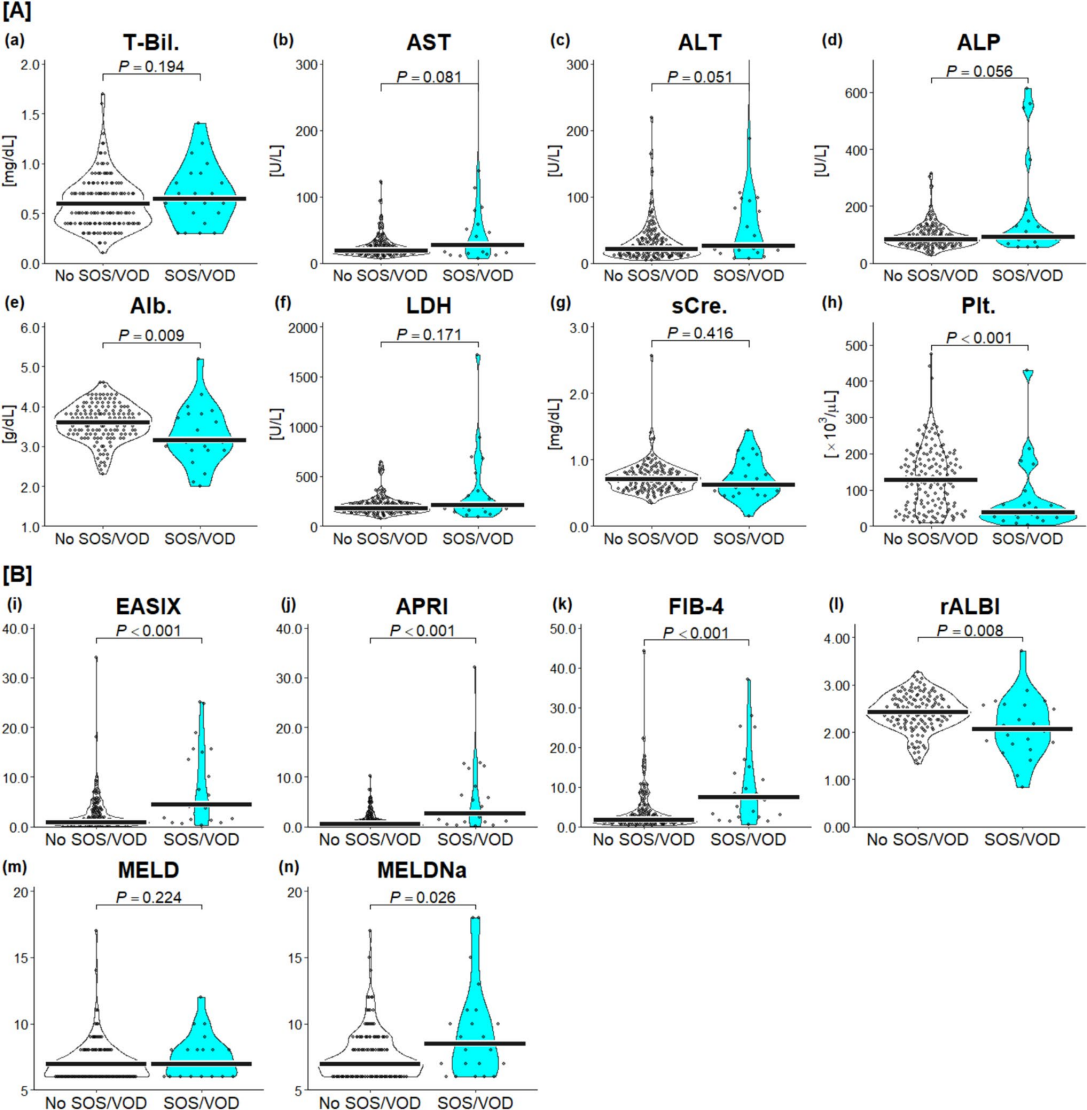
Association of clinical hepatic indices at baseline with SOS/VOD. **A** Individual parameters in blood tests and **B** clinical hepatic indices at baseline are used for the analyses. Dot plots, violin plots, and median values (line) are described and grouped according to eventual “clinical SOS/VOD” development. Objective values are compared using the exact Wilcoxon rank-sum test. Details of the median values (non-SOS/VOD vs. SOS/VOD) are as follows: T-Bil., 0.60 versus 0.65; AST, 19 versus 28; ALT, 22.0 versus 26.5; ALP, 86.0 versus 92.5; Alb., 3.60 versus 3.15; LDH, 185.0 versus 211.5; sCre, 0.71 versus 0.62; Plt., 128 versus 39; EASIX, 1.00 versus 4.45; APRI, 0.6 versus 2.7; FIB-4, 1.80 versus 7.45; rALBI, 2.43 versus 2.08; MELD, 7 versus 7; and MELDNa, 7.0 versus 8.5. Abbreviations: SOS/VOD, sinusoidal obstruction syndrome/veno-occlusive disease; T-Bil., total bilirubin; AST, aspartate aminotransferase; ALT, alanine aminotransferase; ALP, alkaline phosphatase; Alb., albumin; LDH, lactate dehydrogenase; sCre., serum creatinine; Plt., platelets; EASIX, Endothelial Activation and Stress Index; APRI, AST-to-platelet ratio index; FIB-4, Fibrosis-4; rALBI, reversed Albumin-Bilirubin grade; MELD, Model for End-Stage Liver Disease; MELDNa, MELD score and the serum sodium concentration

We present the values of clinical hepatic indices and individual parameters at baseline, grouped by the subsequent SOS/VOD development, in Fig. 1. Albumin levels and platelet counts were significantly associated with SOS/VOD. No significant association was observed with the other individual parameters, including total bilirubin levels. All clinical hepatic indices, except MELD, were significantly associated with SOS/VOD development.

**Fig. 2:**
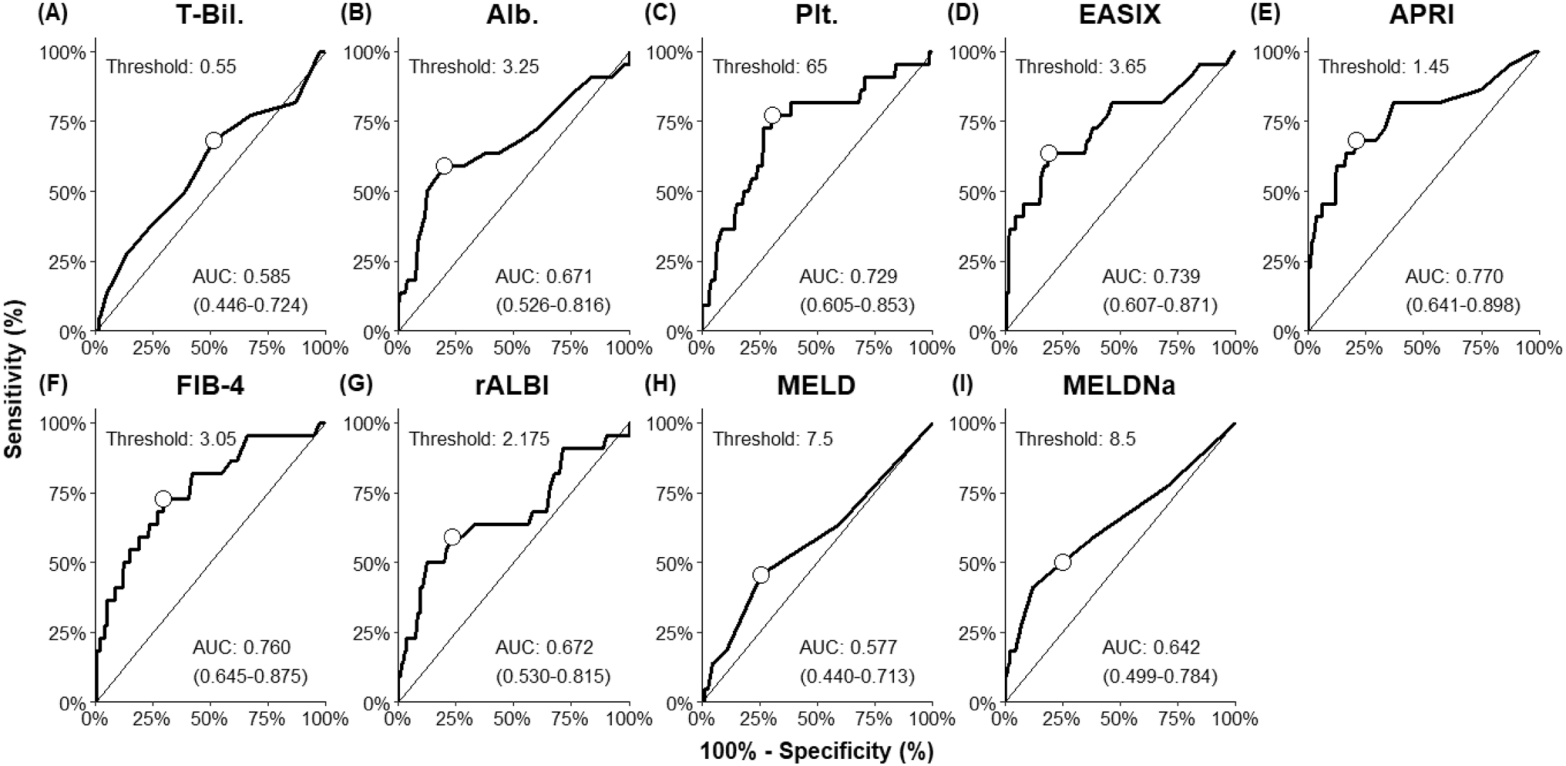
ROC analyses for clinical hepatic indices and individual parameters at baseline. **A**–**C** Individual parameters in blood tests and **D**–**I** clinical hepatic indices at baseline are used for the analyses. The AUC and 95% confidence intervals are shown at the bottom right. Each cutoff threshold is determined as the point closest to the upper-left corner (white circle). Abbreviations: ROC, receiver operating characteristic; AUC, area under the curve; T-Bil., total bilirubin; Alb., albumin; Plt., platelets; EASIX, Endothelial Activation and Stress Index; APRI, AST-to-platelet ratio index; FIB-4, Fibrosis-4; rALBI, reversed Albumin-Bilirubin grade; MELD, Model for End-Stage Liver Disease; MELDNa, MELD score and the serum sodium concentration

**Fig. 3:**
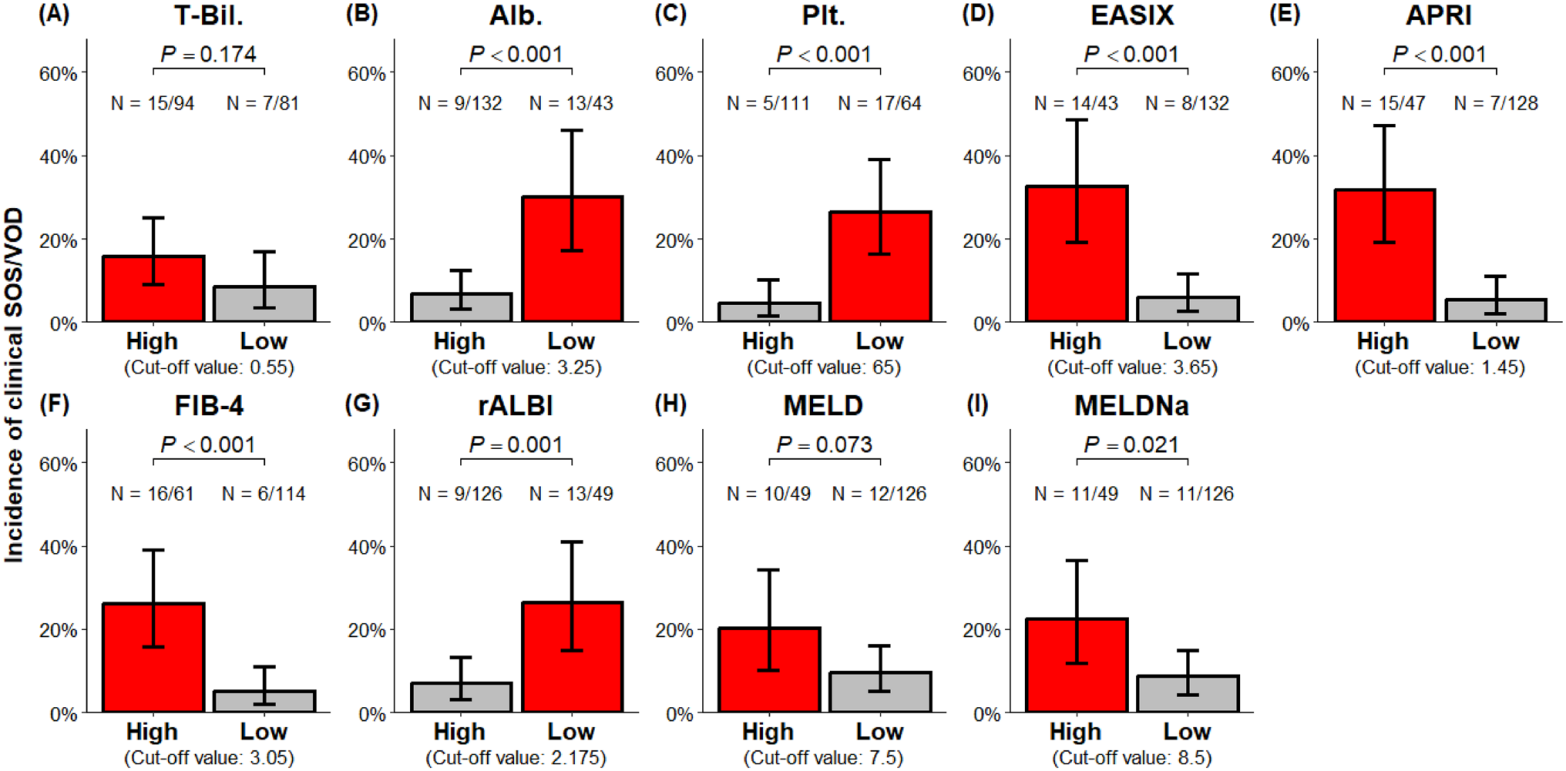
SOS/VOD incidence grouped by clinical hepatic indices at baseline. **A**–**C** Individual parameters in blood tests and **D**–**I** clinical hepatic indices at baseline are used for categorization. The proportions of the eventual “clinical SOS/VOD” incidence and their 95% confidence intervals are described and grouped by high vs. low categorization at baseline. Proportions are compared using the Fisher exact test. The eventual SOS/VOD incidences (high vs. low) are as follows: total bilirubin, 16.0% versus 8.6%; albumin, 6.8% versus 30.2%; platelet, 4.5% versus 26.6%; EASIX, 32.6% versus 6.1%; APRI, 31.9% versus 5.5%; FIB-4, 26.2% versus 5.3%; rALBI, 7.1% versus 26.5%; MELD, 20.4% versus 9.5%; and MELDNa, 22.4% versus 8.7%. Abbreviations: SOS/VOD, sinusoidal obstruction syndrome/veno-occlusive disease; T-Bil., total bilirubin; Alb., albumin; Plt., platelets; EASIX, Endothelial Activation and Stress Index; APRI, AST-to-platelet ratio index; FIB-4, Fibrosis-4; rALBI, reversed Albumin-Bilirubin grade; MELD, Model for End-Stage Liver Disease; MELDNa, MELD score and the serum sodium concentration

Next, we evaluated the predictability of SOS/VOD by focusing on total bilirubin, albumin, platelets, and clinical hepatic indices. We constructed ROC curves and estimated the AUC and 95% confidence interval (Fig. 2). Albumin, platelets, EASIX, APRI, FIB-4, and rALBI demonstrated a significant ability to distinguish SOS/VOD. We compared the AUC of these indices with that of total bilirubin and observed significant superiority with EASIX, APRI, and FIB-4 (*p*-values: EASIX, 0.026; APRI, 0.010; FIB-4, 0.007; ALBI, 0.378; MELD, 0.905; and MELDNa, 0.451). Further, EASIX, APRI, and FIB-4 all exhibited significant superiority over MELD (*p*-values: EASIX, 0.027; APRI, 0.016; FIB-4, 0.015; ALBI, 0.266; and MELDNa, 0.165). No significant differences were observed in the remaining indices (data not shown). Concurrently, we determined the cutoff thresholds and categorized the objective values as high or low using these thresholds. We focused on the low platelets, albumin, and rALBI groups owing to their established association with clinically poor outcomes. We estimated the sensitivity, specificity, positive predictive value, and negative predictive value, as shown in Table 2. Regarding all the variables except for total bilirubin and MELD, significant differences were observed in the eventual SOS/VOD incidences (Fig. 3). Similar analyses focusing on the timing of SOS/VOD onset were performed (Fig. S2, Table S2), suggesting that these indices tended to be associated with particularly “classical SOS/VOD.”

**Table 2:** Diagnostic accuracies of clinical hepatic indexes

	Cut-off value	Sensitivity (%)	Specificity (%)	PPV (%)	NPV (%)
T-Bil	0.55 (mg/dL)	68.2	48.4	16.0	91.4
Alb	3.25 (g/dL)	59.1	80.4	30.2	93.2
Plt	65 (× 10^3^/µL)	77.3	69.3	26.6	95.5
EASIX	3.65	63.6	81.0	32.6	93.9
APRI	1.45	68.2	79.1	31.9	94.5
FIB-4	3.05	72.7	70.6	26.2	94.7
rALBI	2.175	59.1	76.5	26.5	92.9
MELD	7.5	45.5	74.5	20.4	90.5
MELDNa	8.5	50.0	75.2	22.4	91.3

PPV, positive predictive value; NPV, negative predictive value; T-Bil., total bilirubin; Alb., albumin; Plt., platelet; EASIX, Endothelial Activation and Stress Index; APRI, AST (aspartate aminotransferase)-to-platelet ratio index; FIB-4, Fibrosis-4; rALBI, reversed Albumin-Bilirubin grade; MELD, Model for End-Stage Liver Disease; MELDNa, MELD score and the serum sodium concentrate

**Fig. 4:**
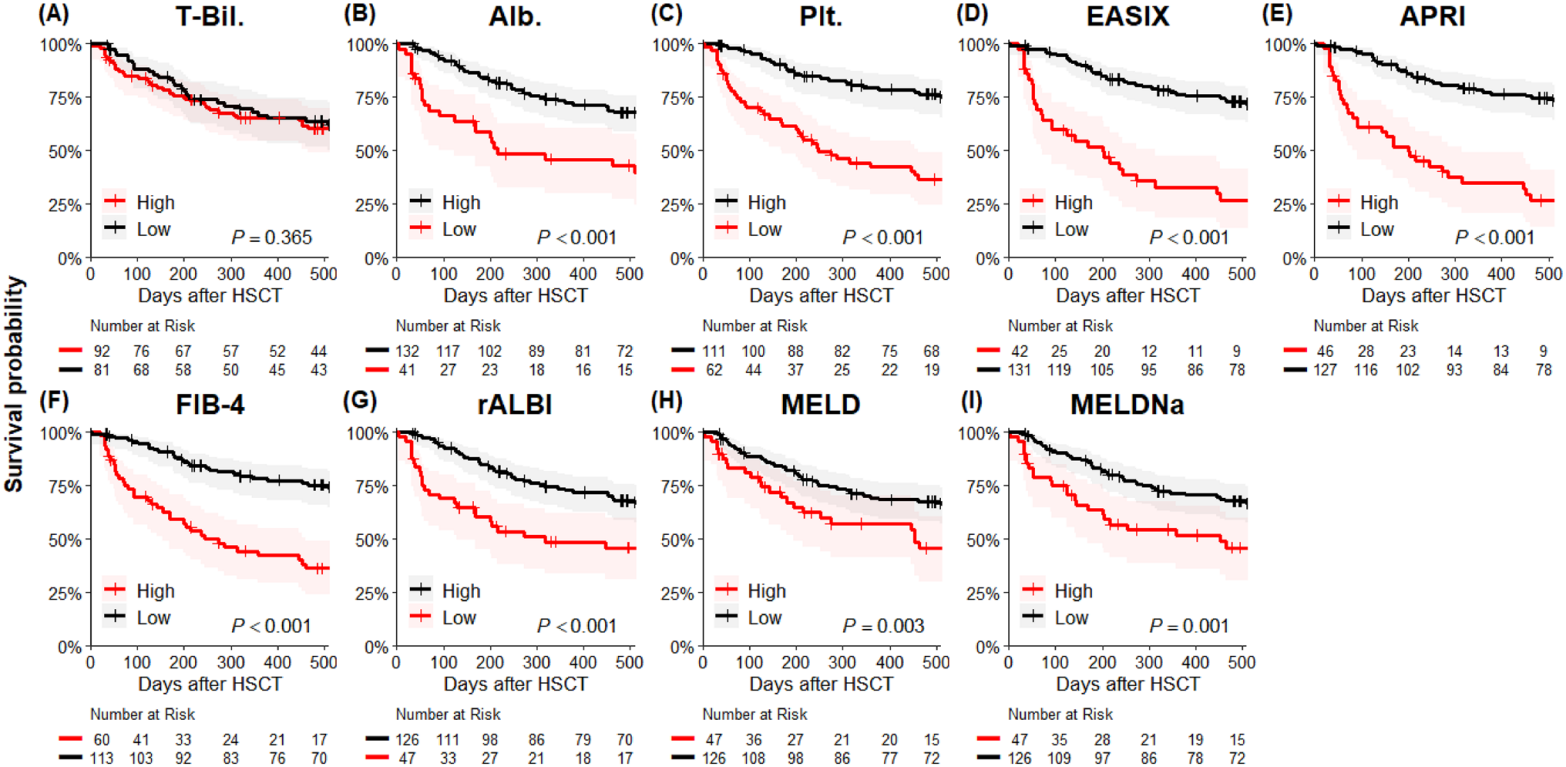
Overall survival after HSCT, grouped by clinical hepatic indices at baseline. **A**–**C** Individual parameters in blood tests and (D–I) clinical hepatic indices at baseline are used for categorization. Overall survival after HSCT for all cases is described and grouped by categorization of clinical hepatic indices and individual parameters at baseline. The 95% confidence intervals are described as fillings. The cutoff values for categorization are as follows: T-Bil., 0.55; Alb., 3.25; Plt., 65; EASIX, 3.65; APRI, 1.45; FIB-4, 3.05; rALBI, 2.175; MELD, 7.5; and MELDNa, 8.5. Abbreviations: HSCT, hematopoietic stem cell transplantation; T-Bil., total bilirubin; Alb., albumin; Plt., platelets; EASIX, Endothelial Activation and Stress Index; APRI, AST to platelet ratio index; FIB-4, Fibrosis-4; rALBI, reversed Albumin-Bilirubin grade; MELD, Model for End-Stage Liver Disease; MELDNa, MELD score and the serum sodium concentration

Using identical values and categorizations, overall survival and TRM after HSCT were stratified as described in Figs. 4 and S3, respectively. All variables, except total bilirubin, were significantly associated with overall survival deterioration. The 100-day overall survival rates (high vs. low) were as follows: total bilirubin, 84.9% vs. 88.4%; albumin, 93.0% vs. 66.4%; platelets, 96.2% vs. 70.0%; EASIX, 59.7% vs. 95.3%; APRI, 61.1% vs. 96.0%; FIB-4, 69.8% vs. 95.5%; rALBI, 93.4% vs. 69.0%; MELD, 81.0% vs. 88.7%; and MELDNa, 74.9% vs. 91.0%. Platelets, EASIX, APRI, FIB-4, and MELD were significantly associated with TRM deterioration.

### Utility of clinical hepatic indices in the impending phase of SOS/VOD

**Fig. 5:**
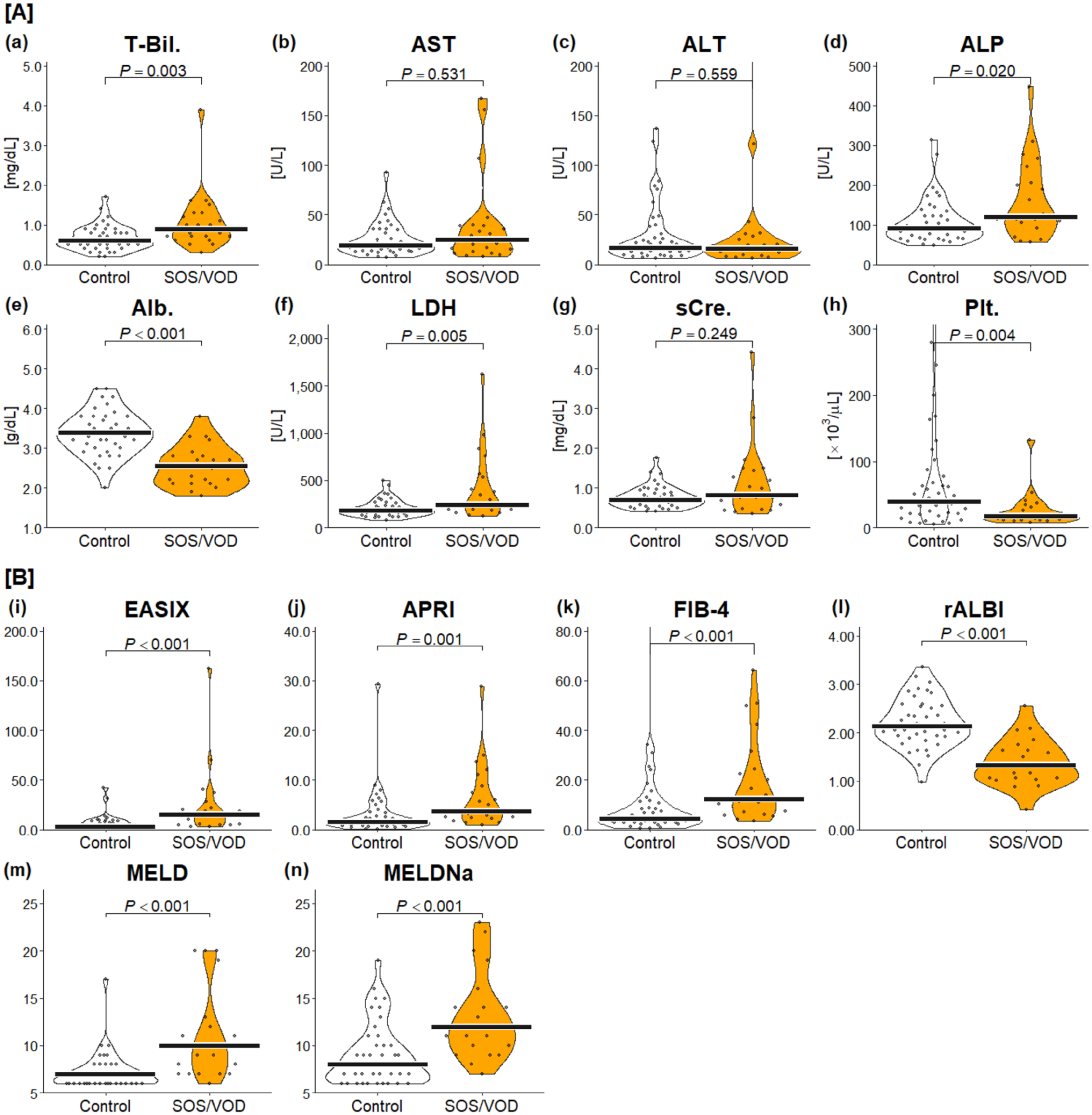
Association of clinical hepatic indices with SOS/VOD, 7 days before diagnosis. **A** Individual parameters in blood tests and **B** clinical hepatic indices 7 days before SOS/VOD diagnosis are used for the analyses. Dot plots, violin plots, and median values (line) within the matched-pair groups, 7 days before SOS/VOD diagnosis are described. Objective values are compared using the exact Wilcoxon rank-sum test. Details of the median values (control vs. SOS/VOD) are as follows: T-Bil., 0.6 versus 0.9; AST, 19 versus 25; ALT, 17.0 versus 16.5; ALP, 91.5 versus 120.0; Alb., 3.40 versus 2.55; LDH, 180.5 versus 245.5; sCre, 0.690 versus 0.815; Plt., 40 versus 18; EASIX, 3.15 versus 15.05; APRI, 1.60 versus 3.65; FIB-4, 4.50 versus 12.35; rALBI, 2.14 versus 1.33; MELD, 7 versus 10; and MELDNa, 8 versus 12. Abbreviations: SOS/VOD, sinusoidal obstruction syndrome/veno-occlusive disease; T-Bil., total bilirubin; Alb., albumin; sCre., serum creatinine; Plt., platelets; EASIX, Endothelial Activation and Stress Index; APRI, AST to platelet ratio index; FIB-4, Fibrosis-4; rALBI, reversed Albumin-Bilirubin grade; MELD, Model for End-Stage Liver Disease; MELDNa, MELD score and the serum sodium concentration

**Fig. 6:**
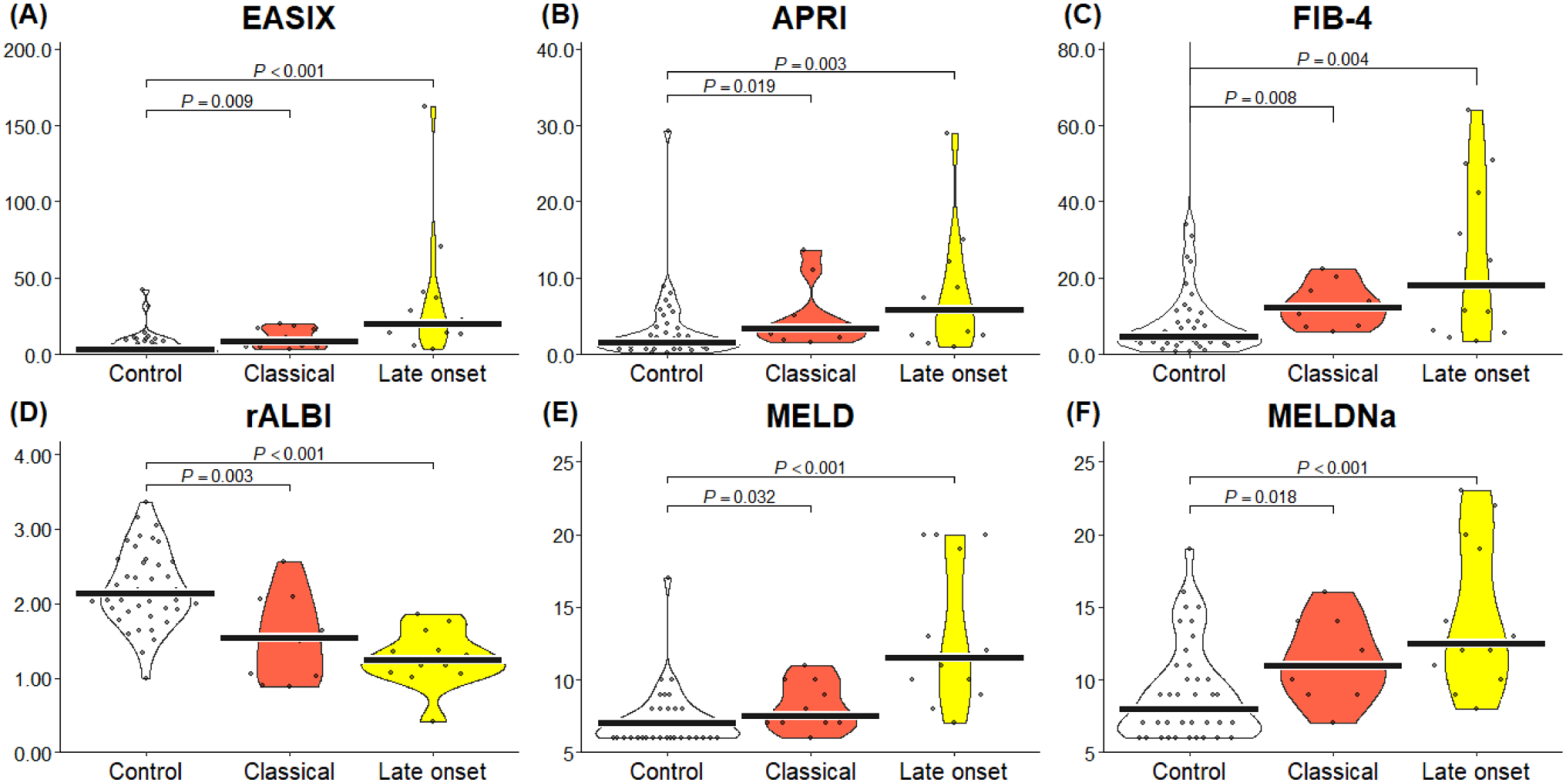
Association of clinical hepatic indices with SOS/VOD, 7 days before diagnosis, focusing on the timing of SOS/VOD diagnosis. Clinical hepatic indices 7 days before SOS/VOD diagnosis are used for the analyses. Dot plots, violin plots, and median values (line) within the matched-pair groups, 7 days before SOS/VOD diagnosis are described. SOS/VOD cases are classified into “classical” or “late onset” SOS/VOD according to the time of diagnosis (within 21 days after HSCT or beyond). Objective values were compared using the exact Wilcoxon rank-sum test. Abbreviations: SOS/VOD, sinusoidal obstruction syndrome/veno-occlusive disease; HSCT, hematopoietic stem cell transplantation; EASIX, Endothelial Activation and Stress Index; APRI, AST to platelet ratio index; FIB-4, Fibrosis-4; rALBI, reversed Albumin-Bilirubin grade; MELD, Model for End-Stage Liver Disease; MELDNa, MELD score and the serum sodium concentration

**Fig. 7:**
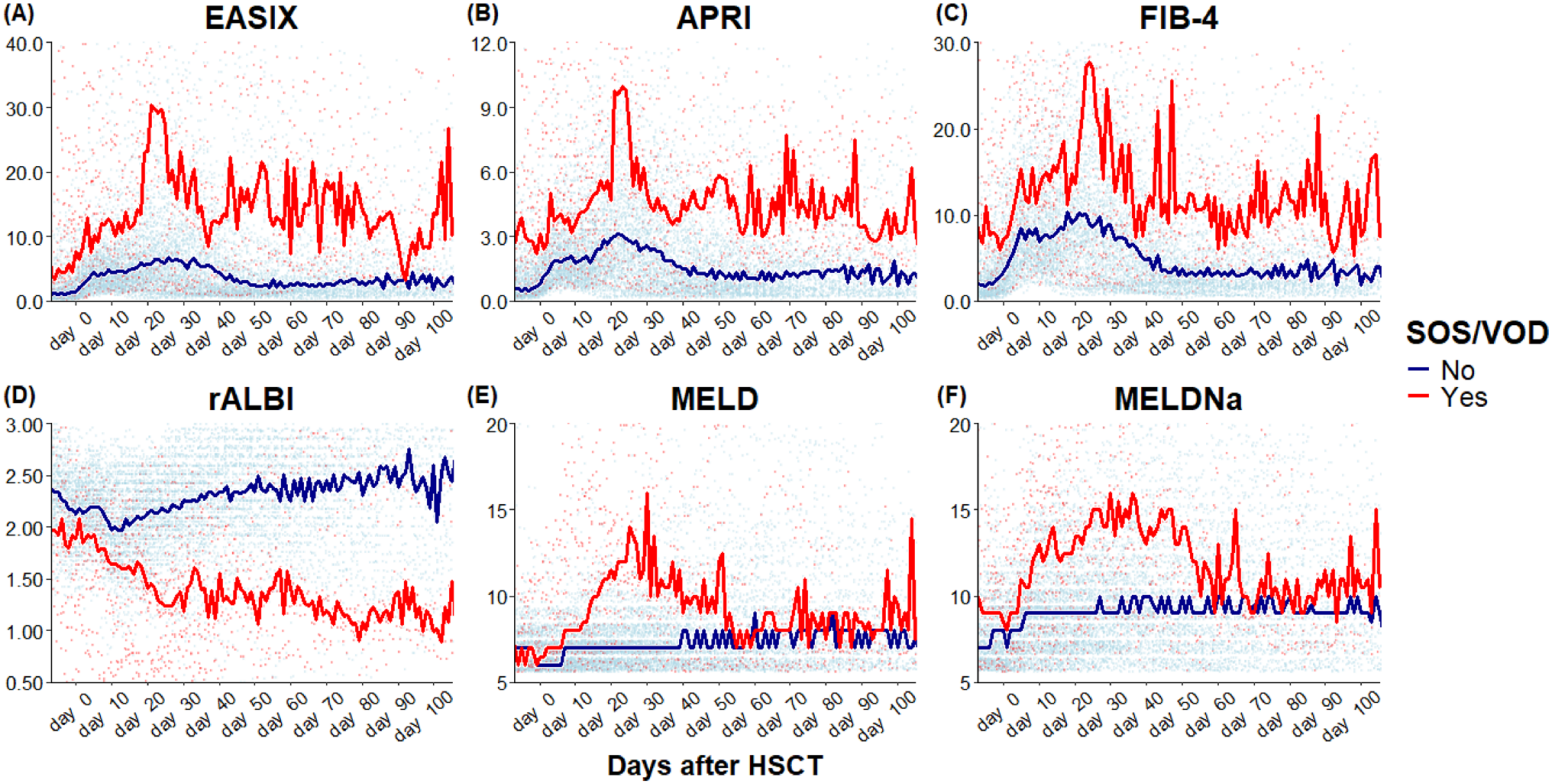
Longitudinal dynamics of clinical hepatic indices after HSCT. **A** EASIX, **B** APRI, **C** FIB-4, **D** rALBI, **E** MELD, and **F** MELDNa. The dynamics of the median values of clinical hepatic indices after HSCT are described and grouped according to SOS/VOD development, including all cases. Retroactive data substitution is not allowed exceptionally in this figure. Detailed values are plotted as pale dots. Abbreviations: SOS/VOD, sinusoidal obstruction syndrome/veno-occlusive disease; HSCT, hematopoietic stem cell transplantation; EASIX, Endothelial Activation and Stress Index; APRI, AST to platelet ratio index; FIB-4, Fibrosis-4; rALBI, reversed Albumin-Bilirubin grade; MELD, Model for End-Stage Liver Disease; MELDNa, MELD score and the serum sodium concentration

We sampled 66 cases from the non-SOS/VOD group as controls. The standardized differences in the matching variables between the control and SOS/VOD groups were below 0.10–0.15, indicating an acceptable balance (Table S3). Comparing SOS/VOD cases with controls, all clinical hepatic indices were significantly associated with SOS/VOD on the day of diagnosis (Fig. S4). Similarly, 7 days before diagnosis, all hepatic indices were significantly associated with SOS/VOD (Fig. 5). In contrast, several individual parameters were not significantly associated with SOS/VOD, 7 days before diagnosis. Then, we performed similar analyses focusing on the timing of SOS/VOD onset (Fig. 6), demonstrating stronger associations between these indices measured 7 days before diagnosis and “late onset SOS/VOD.” Finally, we illustrated the longitudinal dynamics of clinical hepatic indices after HSCT, including all cases in our cohort (Fig. 7). All hepatic indices tended to worsen between days 10 and 30 and gradually improved thereafter. The dynamics of the indices varied between cases with and without SOS/VOD.

## Discussion

In this study, most clinical hepatic indices at baseline, in addition to albumin levels and platelet counts, were significantly associated with SOS/VOD development. AUC analyses and the observed SOS/VOD incidence indicated that albumin, platelets, EASIX, APRI, FIB-4, and rALBI at baseline had significant predictive values. Notably, EASIX, APRI, and FIB-4 showed superior predictive performance compared to total bilirubin and MELD. Therefore, these three indices were particularly useful in predicting SOS/VOD even before the initiation of conditioning. These findings are consistent with previous reports regarding albumin [22], EASIX [12, 13], and FIB-4 [23] at baseline. In addition, all clinical hepatic indices significantly predicted poor overall survival in this study. The results indicate their utility in planning treatment strategies for allogeneic HSCT, including decisions regarding conditioning intensity.

The findings at baseline may be less suitable for assessing clinical events occurring late after HSCT, particularly in cases of “late onset SOS/VOD” (defined as ≥ 22 days after HSCT [7, 24]). Thus, we examined the behavior of hepatic indices in the post-HSCT setting. At the time of SOS/VOD diagnosis, individual parameters substantially differed between the SOS/VOD and control group, as expected. Hepatic indices also significantly differed at that time, suggesting their utility in diagnosing even “late onset SOS/VOD.” Furthermore, these indices exhibited significant differences as early as 7 days before SOS/VOD diagnosis. This finding contrasted with the pattern observed for individual parameters. Several parameters did not reach statistical significance, while others showed minor differences with limited clinical relevance (such as median total bilirubin: 0.6 vs. 0.9 mg/dL). These results indicate that clinical hepatic indices in the post-HSCT setting are useful for detecting impending SOS/VOD early and may offer superior utility compared with individual parameters. Importantly, these indices measured 7 days before diagnosis were more strongly associated with “late onset SOS/VOD” than with “classical SOS/VOD” (Fig. 6). This may reflect the more gradual changes in patients’ clinical conditions during the late phase of HSCT. These findings suggest that these indices may also be valuable for early SOS/VOD detection in outpatient settings. More detailed behavior of these indices remains poorly understood, as they fluctuate markedly on a daily basis, as shown in Fig. 7. Determining their appropriate cutoff thresholds in the post-HSCT setting was also challenging using conventional methods such as ROC analysis. Therefore, trend-based evaluation, as we demonstrated in this study, will be more clinically informative than reliance on single time point measurements. Further studies using larger datasets would be necessary to better understand the time-dependent dynamics of these indices.

Clinical hepatic indices are highly beneficial, as they can be calculated quickly, repeated daily, and do not require specialized techniques. This characteristic presents a clear advantage over the other tests, such as biomarker-based assays, which are presumably SOS/VOD-specific [25–27]. Particularly, these indices can be easily assessed in the very early phase following HSCT, when invasive procedures are difficult to perform. In addition to these advantages, we observed a relatively high specificity for these indices in this study. However, notably, not all the reference standard cases in this study were confirmed as SOS/VOD by histopathology (“proven SOS/VOD”). Other noninvasive approaches may serve as complementary tools, including novel ultrasound techniques, HokUS-10/6 [28, 29] and/or elastography [30, 31].

Our results underscore the importance of platelet count in SOS/VOD evaluation. Importantly, the formulas for EASIX, APRI, and FIB-4 incorporated platelet counts, all demonstrating significant predictive values at baseline in this study. The association between thrombocytopenia and SOS/VOD has long been recognized [32, 33]. Although the detailed pathophysiology remains incompletely understood, SOS/VOD is considered to originate from endothelial injury, leading to thrombotic pathway activation and platelet aggregation in the extravascular space [34, 35]. This pathogenesis explains why thrombocytopenia is one of the earliest SOS/VOD manifestations [36, 37]. Accordingly, platelet count monitoring is crucial for early detection. Both the pediatric EBMT criteria [38] and the proposed “Cairo criteria” [39, 40] for SOS/VOD include thrombocytopenia as a diagnostic parameter. Nevertheless, it is challenging to implicate thrombocytopenia in SOS/VOD diagnosis, owing to its common occurrence in the early post-HSCT phase. Moreover, careful consideration of the underlying disease status is required, as thrombocytopenia may also reflect bone marrow suppression related to the primary disease; indeed, some patients with SOS/VOD in this study already had progressive disease at baseline. From this perspective, the use of clinical hepatic indices may be beneficial in evaluating concealing pathologies. In addition, serum albumin levels, which comprise the formula of ALBI, appear to be important. However, compared to platelet count, the role of albumin in diagnosing SOS/VOD has not been thoroughly investigated. Further studies are necessary, particularly from a pathophysiological perspective. In contrast, total bilirubin at baseline did not demonstrate significant predictive value despite its essential role in diagnosing SOS/VOD. This finding is consistent with the current knowledge that bilirubin elevation occurs at a relatively later phase in patients with SOS/VOD [8, 24]. A similar trend was observed for MELD and MELDNa, suggesting that these indices were more applicable during the progression of SOS/VOD rather than at baseline. Consequently, it may be appropriate to use each hepatic index depending on the clinical course. We believe that properly integrating various types of clinical information is the most effective approach for early and accurate SOS/VOD detection.

Our study had some limitations. First, it was conducted at a single institution with a relatively small number of patients, which precluded certain analyses, such as the association between the hepatic indices and SOS/VOD severity or the efficacy of defibrotide. Second, we did not perform multivariable analyses because of the limited number of events and the strong correlations among these indices resulting from shared parameters. Consequently, some questions remain unresolved, including which index is most appropriate for clinical use. Larger sample sizes are required to perform these analyses with stable estimation. Third, overfitting was an unavoidable issue in the study design. Larger external validation studies are warranted to address these limitations. Our study has several strengths despite these limitations. To the best of our knowledge, this is the first report to investigate the behavior of several hepatic indices in SOS/VOD after HSCT, including ALBI and MELDNa. We further examined the detailed dynamics of hepatic indices in a post-HSCT setting.

In conclusion, clinical hepatic indices, namely EASIX, APRI, FIB-4, ALBI, MELD, and MELDNa, are useful for predicting and facilitating early SOS/VOD diagnosis, irrespective of the phase in allogeneic HSCT. EASIX, APRI, and FIB-4 are considered particularly useful for predicting SOS/VOD before the initiation of conditioning. Further studies are warranted to optimize the clinical application of these indices.

## Supplementary Information

Below is the link to the electronic supplementary material.

Supplementary file1 (DOCX 433 kb)

## Data Availability

Data supporting the findings of this study shall be made available from the corresponding author upon a reasonable request.
