# Supplementary material for: Clinical hepatic indices serve as predictive markers for sinusoidal obstruction syndrome after allogeneic HSCT
Source: Int J Hematol. 2026 Mar 16;124(1):114–25. doi: 10.1007/s12185-026-04191-5 (PMC13319434; doi:10.1007/s12185-026-04191-5)
Supplement: Supplementary file 1 — Supplementary file1 (DOCX 433 kb) [file 12185_2026_4191_MOESM1_ESM.docx]

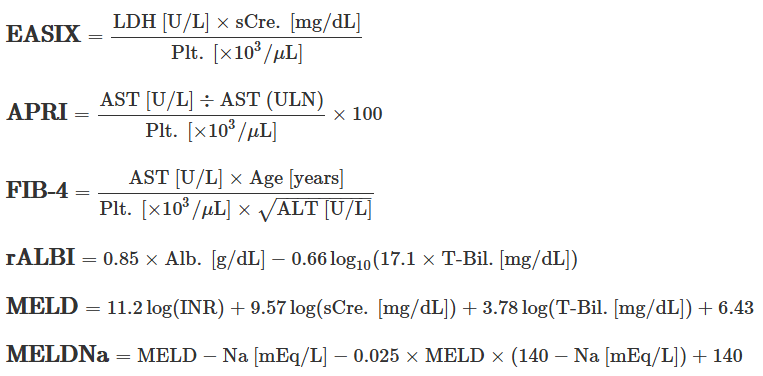


**Figure S1. Calculation formulas of clinical hepatic indices**

The upper limit of normal for AST is 30 U/L at our institute. For the calculation of MELD, the base of natural logarithms is used, with a lower limit of 1.0 for all variables and an upper limit of 4.00 for serum creatinine. If the patient is undergoing dialysis at the objective day, the serum creatinine level is equated with 4.00 mg/dL. To calculate MELDNa, the serum sodium concentration is bound between 125 and 140 mEq/L. Both MELD and MELDNa are rounded to the nearest integer.

Abbreviations: LDH, lactate dehydrogenase; sCre., serum creatinine; Plt., platelets; AST, aspartate aminotransferase; ALT, alanine aminotransferase; Alb., albumin; T-Bil., total bilirubin; INR, prothrombin time-international normalized ratio; ULN, upper limit of normal; EASIX, Endothelial Activation and Stress Index; APRI, AST to platelet ratio index; FIB-4, Fibrosis-4; rALBI, reversed Albumin-Bilirubin grade; MELD, Model for End-Stage Liver Disease; MELDNa, MELD score and the serum sodium concentration.


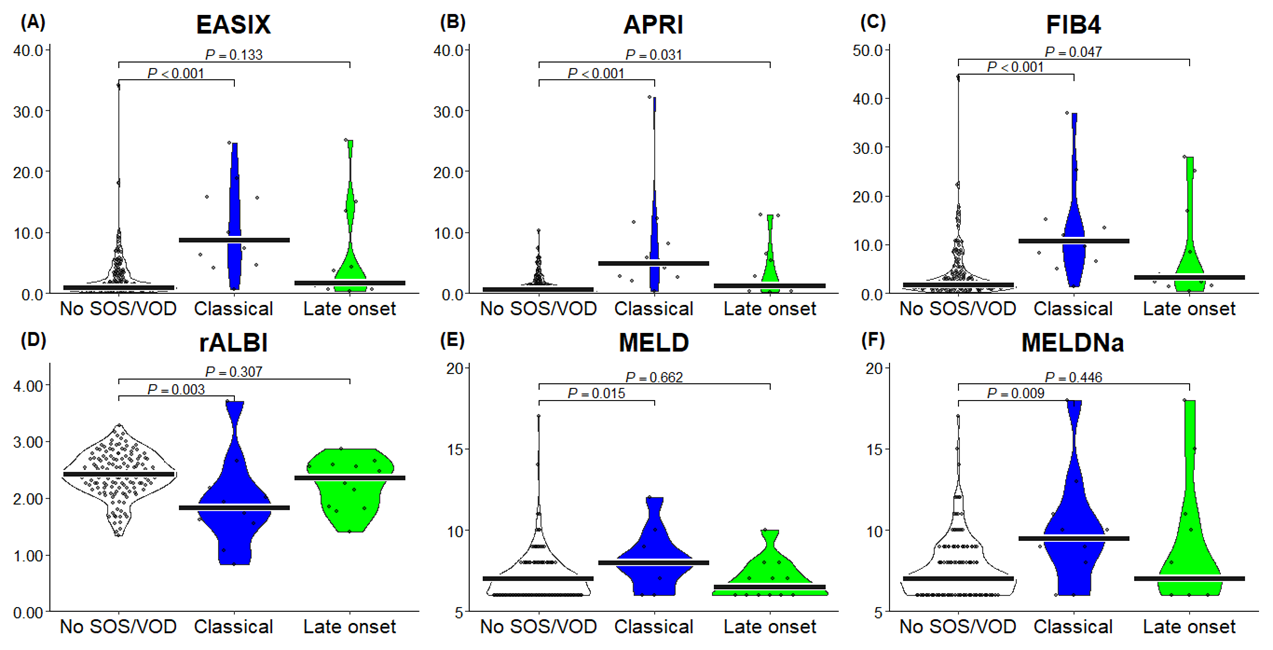


**Figure S2. Association of clinical hepatic indices at baseline with SOS/VOD, focusing on developing time**

Clinical hepatic indices at baseline are used for the analyses. Dot plots, violin plots, and median values (line) are described and grouped according to eventual “clinical SOS/VOD” development. SOS/VOD cases are classified into “classical” or “late onset” SOS/VOD by diagnosed time (within days 21 after HSCT or not). Objective values are compared using the exact Wilcoxon rank-sum test.

Abbreviations: SOS/VOD, sinusoidal obstruction syndrome/veno-occlusive disease; HSCT, hematopoietic stem cell transplantation; EASIX, Endothelial Activation and Stress Index; APRI, AST to platelet ratio index; FIB-4, Fibrosis-4; rALBI, reversed Albumin-Bilirubin grade; MELD, Model for End-Stage Liver Disease; MELDNa, MELD score and the serum sodium concentration.


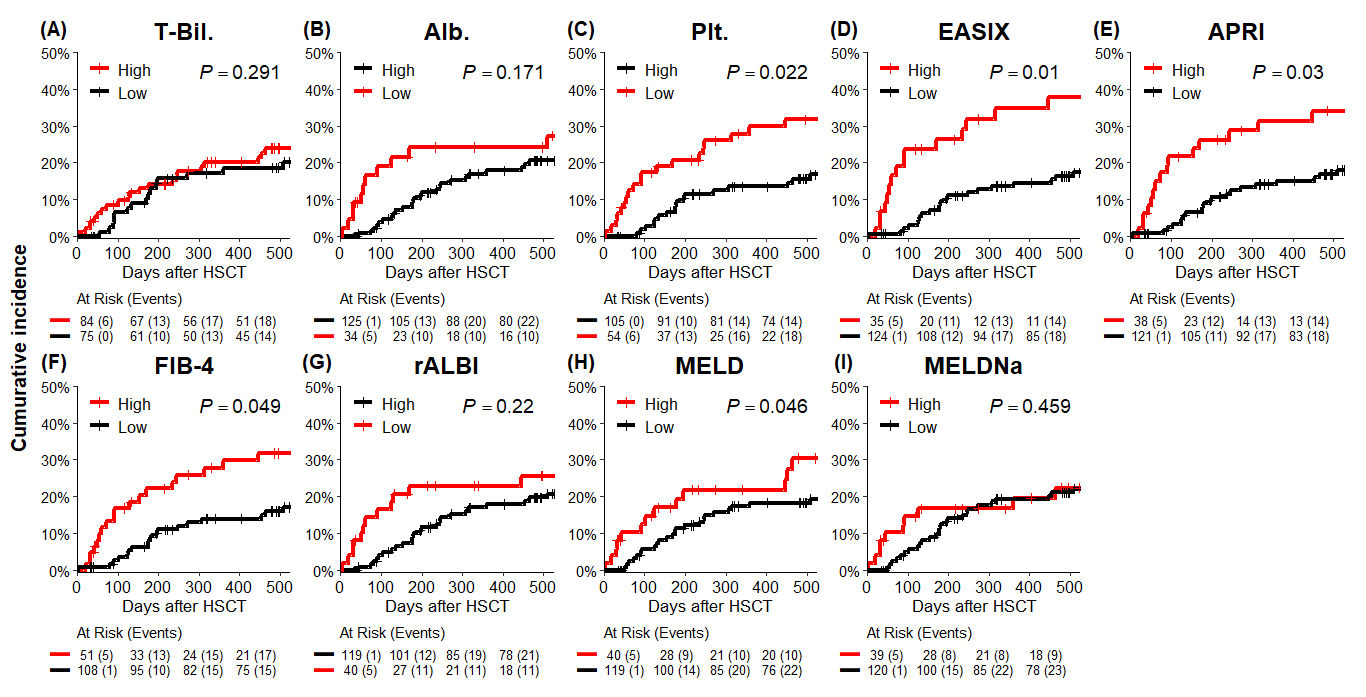


**Figure S3. Transplantation-related mortality after HSCT**

(A–C) Individual parameters in blood tests and (D–I) clinical hepatic indices at baseline are used for categorization.

Transplantation-related mortality is described using the Fine–Gray method, considering relapse-related death as a competing risk. Categorization is based on the baseline values of the variables. The cut-off values for categorization are as follows: T-Bil., 0.55; Alb., 3.25; Plt., 65; EASIX, 3.65; APRI, 1.45; FIB-4, 3.05; rALBI, 2.175; MELD, 7.5; and MELDNa, 8.5.

Abbreviations: HSCT, hematopoietic stem cell transplantation; T-Bil., total bilirubin; Alb., albumin; Plt., platelets; EASIX, Endothelial Activation and Stress Index; APRI, AST to platelet ratio index; FIB-4, Fibrosis-4; rALBI, reversed Albumin-Bilirubin grade; MELD, Model for End-Stage Liver Disease; MELDNa, MELD score and the serum sodium concentration.


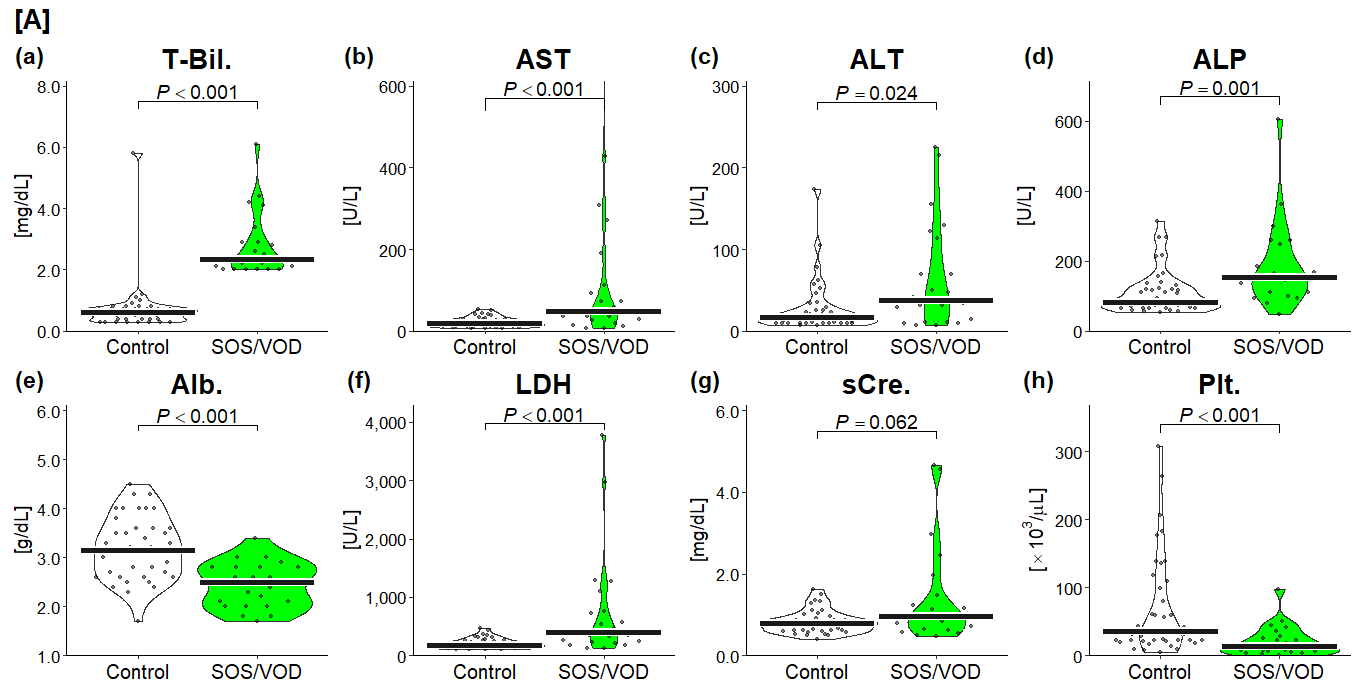

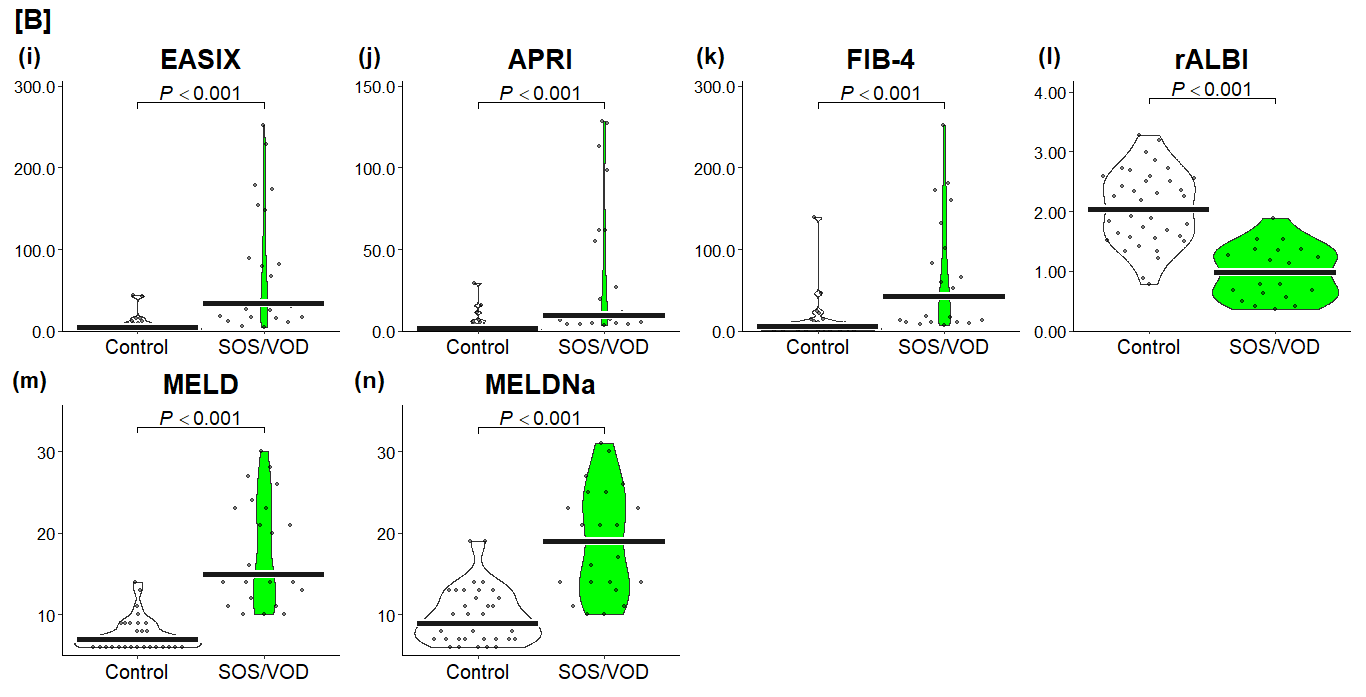


**Figure S4. Association of clinical hepatic indices with SOS/VOD on the day of diagnosis**

(A) Individual parameters in blood tests and (B) clinical hepatic indices on the day of SOS/VOD diagnosis are used for the analyses.

Dot plots, violin plots, and median values (line) within the matched-pair groups on the day of SOS/VOD diagnosis are described. Details of the median values (control vs. SOS/VOD) are as follows: T-Bil., 0.60 vs. 2.35; AST, 20.0 vs. 47.5; ALT, 17.0 vs. 37.5; ALP, 83.5 vs. 152.5; Alb., 3.15 vs. 2.50; LDH, 181 vs. 391; sCre, 0.795 vs. 0.970; Plt., 35.5 vs. 13.0; EASIX, 4.2 vs. 33.7; APRI, 1.5 vs. 9.9; FIB-4, 5.35 vs. 42.50; rALBI, 2.035 vs. 0.980; MELD, 7 vs. 15; and MELDNa, 9 vs. 19.

Abbreviations: SOS/VOD, sinusoidal obstruction syndrome/veno-occlusive disease; T-Bil., total bilirubin; Alb., albumin; sCre., serum creatinine; Plt., platelet; EASIX, Endothelial Activation and Stress Index; APRI, AST to platelet ratio index; FIB-4, Fibrosis-4; rALBI, reversed Albumin-Bilirubin grade; MELD, Model for End-Stage Liver Disease; MELDNa, MELD score and the serum sodium concentration.

| **Patient No.** | **Age at HSCT** | **Sex** | **Datailed diagnosis of SOS/VOD** | **Diagnosed days after HSCT** | **T-Bil. [mg/dL]** | **Painful hepatomegaly** | **Weight gain** | **Ascites** |
| --- | --- | --- | --- | --- | --- | --- | --- | --- |
| 5 | 41 | Male | Classical | 20 | 2.1 | - | + | + |
| 13 | 60 | Male | Late onset | 126 | 2.3 | - | + | + |
| 29 | 53 | Female | Late onset | 25 | 4.2 | - | + | + |
| 34 | 37 | Female | Late onset | 187 | 2.5 | - | + | + |
| 46 | 28 | Female | Classical | 6 | 4.4 | - | + | + |
| 55 | 65 | Male | Late onset | 25 | 2.1 | - | + | + |
| 93 | 53 | Female | Classical | 20 | 2.2 | - | + | + |
| 95 | 36 | Male | Classical | 12 | 2.0 | + | + | + |
| 96 | 62 | Male | Late onset | 79 | 4.1 | - | + | + |
| 97 | 52 | Male | Late onset | 41 | 6.1 | - | + | + |
| 109 | 54 | Male | Classical | 6 | 2.2 | - | + | + |
| 117 | 54 | Male | Classical | 20 | 2.0 | - | + | + |
| 120 | 32 | Male | Late onset | 238 | 2.4 | - | + | + |
| 124 | 38 | Female | Classical | 14 | 2.0 | + | + | - |
| 128 | 57 | Male | Classical | 2 | 2.9 | - | + | + |
| 159 | 48 | Male | Late onset | 56 | 2.0 | + | + | + |
| 166 | 64 | Female | Late onset | 502 | 2.8 | - | + | + |
| 169 | 37 | Female | Late onset | 34 | 2.6 | - | + | + |
| 82 | 55 | Female | Proven | 11 | 2.9 | + | + | - |
| 85 | 53 | Female | Proven | 73 | 2.0 | + | + | + |
| 91 | 38 | Male | Proven | 22 | 2.0 | + | + | + |
| 106 | 37 | Female | Proven | 6 | 3.4 | - | + | + |
| 43 | 53 | Male | Other diagnosis | 117 | 3.3 | + | - | + |
| 83 | 47 | Female | Other diagnosis | 303 | 2.8 | - | + | + |
| 155 | 65 | Female | Other diagnosis | 97 | 2.0 | + | - | + |

**Table S1. Detailed characteristics of SOS/VOD patients at the diagnosis**

Abbreviations: SOS/VOD, sinusoidal obstruction syndrome/veno-occlusive disease; HSCT, hematopoietic stem cell transplantation; T-Bil., total bilirubin.

| **Classical SOS/VOD** | | | | | |
| --- | --- | --- | --- | --- | --- |
|  | Cut-off value | Sensitivity (%) | Specificity (%) | PPV (%) | NPV (%) |
| T-Bil. | 0.65 (mg/dL) | 70.0 | 60.8 | 10.4 | 96.9 |
| Alb. | 3.25 (g/dL) | 80.0 | 80.4 | 21.1 | 98.4 |
| Plt. | 65 (x10^3^/μL) | 90.0 | 69.3 | 16.1 | 99.1 |
| EASIX | 4.00 | 90.0 | 83.0 | 25.7 | 99.2 |
| APRI | 1.95 | 90.0 | 83.7 | 26.5 | 99.2 |
| FIB-4 | 4.80 | 90.0 | 81.0 | 23.7 | 99.2 |
| rALBI | 2.175 | 80.0 | 76.5 | 18.2 | 98.3 |
| MELD | 7.5 | 70.0 | 74.5 | 15.2 | 97.4 |
| MELDNa | 8.5 | 70.0 | 75.2 | 15.6 | 97.5 |
| **Late onset SOS/VOD** | | | | | |
|  | Cut-off value | Sensitivity (%) | Specificity (%) | PPV (%) | NPV (%) |
| T-Bil. | 0.55 (mg/dL) | 66.7 | 48.4 | 9.2 | 94.9 |
| Alb. | 3.25 (g/dL) | 41.7 | 80.4 | 14.3 | 94.6 |
| Plt. | 59 (x10^3^/μL) | 66.7 | 73.2 | 16.3 | 96.6 |
| EASIX | 1.25 | 75.0 | 53.6 | 11.3 | 96.5 |
| APRI | 0.95 | 75.0 | 63.4 | 13.8 | 97.0 |
| FIB-4 | 2.25 | 75.0 | 58.2 | 12.3 | 96.7 |
| rALBI | 2.255 | 50.0 | 66.7 | 10.5 | 94.4 |
| MELD | 6.5 | 50.0 | 59.5 | 8.8 | 93.8 |
| MELDNa | 9.5 | 33.3 | 88.2 | 18.2 | 94.4 |

**Table S2. Diagnostic accuracies of clinical hepatic indices, focusing on developing time**

Abbreviations: SOS/VOD, sinusoidal obstruction syndrome/veno-occlusive disease; PPV, positive predictive value; NPV, negative predictive value; T-Bil., total bilirubin; Alb., albumin; Plt., platelet; EASIX, Endothelial Activation and Stress Index; APRI, AST (aspartate aminotransferase) to platelet ratio index; FIB-4, Fibrosis-4; rALBI, reversed Albumin-Bilirubin grade; MELD, Model for End-Stage Liver Disease; MELDNa, MELD score and the serum sodium concentration.

|  | **Control** | **SOS/VOD** | **Std. diff.** |
| --- | --- | --- | --- |
| **N** | 44 | 22 |  |
| **Age, median (range)** | 47 (27–65) | 52 (28–65) | 0.029 |
| **Sex (%)** |  |  | <0.001 |
| Male/Female | 24/20 (54.5/45.5) | 12/10 (54.5/45.5) |  |
| **HCT-CI (%)** |  |  | 0.125 |
| 0/1–2/>3 | 3/12/29 (6.8/27.3/65.9) | 2/5/15 (9.1/22.7/68.2) |  |
| **HLA genotype mismatch (%)** |  |  | 0.045 |
| Yes/No | 21/23 (47.7/52.3) | 11/11 (50.0/50.0) |  |
| **Conditioning (%)** |  |  | <0.001 |
| MAC/RIC | 20/24 (45.5/54.5) | 10/12 (45.5/54.5) |  |

**Table S3. Characteristics of matched-pair groups**

Abbreviations: SOS/VOD, sinusoidal obstruction syndrome/veno-occlusive disease; std. diff., standardized difference; HCT-CI, hematopoietic cell transplant-specific comorbidity index; HLA, human leukocyte antigen; MAC, myeloablative conditioning; RIC, reduced-intensity conditioning.
